# Association of Vericiguat with Improvement in Functional Abilities and Comprehensive Geriatric Assessment in Elderly Patients with Worsening Heart Failure

**DOI:** 10.3390/ph19030466

**Published:** 2026-03-12

**Authors:** Giuseppe Armentaro, Maria Rosangela Scarcelli, Giandomenico Severini, Carlo Alberto Pastura, Velia Cassano, Francesco Maruca, Laura Francesca Marincola, Gianluca Cortese, Valentino Condoleo, Sofia Miceli, Raffaele Maio, Maurizio Volterrani, Cristiana Vitale, Giuseppe Massimo Claudio Rosano, Angela Sciacqua

**Affiliations:** 1 Geriatrics Division, “Renato Dulbecco” University Hospital of Catanzaro, 88100 Catanzaro, Italy; giandomenicoseverini@gmail.com (G.S.); condoleovalentino@gmail.com (V.C.); sofy.miceli@libero.it (S.M.); raf_maio@yahoo.it (R.M.); sciacqua@unicz.it (A.S.); 2 Department of Medical and Surgical Sciences, University Magna Græcia of Catanzaro, 88100 Catanzaro, Italy; mariarosangelascarcelli@gmail.com (M.R.S.); carloalbertopastura@gmail.com (C.A.P.); velia.cassano@unicz.it (V.C.); francesco.maruca28@gmail.com (F.M.); laurmar@hotmail.it (L.F.M.); cortesegianluca97@gmail.com (G.C.); 3 Istituto di Ricovero e Cura a Carattere Scientifico (IRCCS), San Raffaele, 00163 Roma, Italy; maurizio.volterrani@sanraffaele.it (M.V.); cristiana.vitale@gmail.com (C.V.); giuseppe.rosano@gmail.com (G.M.C.R.); 4 Department of Human Sciences and Promotion of Quality of Life, San Raffaele Open University of Rome, 00166 Rome, Italy

**Keywords:** worsening heart failure, vericiguat, elderly, comprehensive geriatric assessment

## Abstract

**Background**: Elderly patients with heart failure with reduced ejection fraction (HFrEF) who experience worsening heart failure (wHF) remain at high residual risk despite optimal medical therapy (OMT), and data on cognitive function and comprehensive geriatric assessment (CGA) in this setting are lacking. This study evaluated the association between 12-month treatment with vericiguat and changes in cardiac, functional and geriatric parameters in elderly patients with recent wHF. **Methods and results**: In this single-center prospective observational study, 55 patients (45 men, mean age 76.4 ± 5.1 years) with HFrEF on OMT and a recent episode of wHF were treated with vericiguat and followed for 12 months. Clinical assessment, CGA and echocardiography including speckle-tracking were performed at baseline, 6, and 12 months. At 12 months, the mean vericiguat dose was 5.5 ± 2.9 mg/day. NT-proBNP levels decreased from 980 (467–2106) to 654 (274–1762) pg/mL (*p* < 0.0001), while left ventricular ejection fraction increased from 36.8 ± 3.1% to 43.4 ± 5.7% (*p* < 0.0001). Global longitudinal strain improved from −9.2 ± 1.7% to −11.5 ± 2.1% (*p* = 0.008), with parallel improvements in right ventricular function and pulmonary pressures. Cognitive performance improved (MMSE 25.1 ± 1.7 to 26.2 ± 2.1 points, *p* < 0.0001), as did depressive symptoms (GDS 7.8 ± 2.0 to 5.4 ± 1.6 points, *p* < 0.0001), physical performance (SPPB 6.7 ± 1.1 to 8.4 ± 0.9 points, *p* < 0.0001), and gait speed (0.70 ± 0.10 to 0.83 ± 0.06 m/s, *p* < 0.0001). Conley score decreased from 5.2 ± 2.3 to 2.4 ± 1.8 points (*p* < 0.0001), suggesting a lower risk of falls. Loop diuretic and MRA use were significantly reduced during follow-up. **Conclusions**: In this elderly HFrEF cohort with recent wHF on contemporary OMT, 12-month treatment with vericiguat was associated with consistent improvements in cardiac structure and function, biomarkers, and multidimensional geriatric status. These hypothesis-generating findings support the integration of CGA into future controlled studies of vericiguat in frail older patients with HFrEF. Given the observational design and lack of a control group, causal inference is not possible.

## 1. Introduction

Heart failure (HF) is a complex clinical syndrome, characterized by typical symptoms (dyspnea, fatigue, peripheral edema) and clinical signs (pulmonary congestion, increased jugular venous pressure, basal crackles), caused by a structural and/or functional abnormality of the heart that impairs the ability of the ventricle to fill or eject blood adequately to meet the body’s needs [1]. The recent universal definition of HF requires three fundamental criteria: the presence of symptoms and/or clinical signs of heart failure, objective evidence of cardiac dysfunction (systolic and/or diastolic), and, in doubtful cases, a positive response to specific HF therapy [1].

Many patients, in the natural history of the disease, experience episodes of exacerbation and worsening of heart failure (wHF) that often require hospitalization or outpatient procedures involving intravenous diuretic therapy, with a significant increase in the risk of mortality [2,3]. Since each episode of exacerbation correlates with worsening prognosis [2], the main challenge in the management of patients with HF is the early recognition and timely treatment of wHF.

Despite notable therapeutic advances in heart failure with reduced ejection fraction (HFrEF), this syndrome remains associated with high morbidity and mortality. The introduction of cornerstone therapies, such as ACE inhibitors, beta-blockers, mineralocorticoid receptor antagonists (MRAs), sacubitril/valsartan (ARNI), and, more recently, sodium-glucose cotransporter-2 inhibitors (SGLT2-i), has significantly improved the clinical outcome of patients [3,4]. However, even in optimally treated patients, a residual risk of cardiovascular adverse events and mortality persists.

Residual risk in HFrEF refers to the proportion of adverse events, such as exacerbations, hospitalizations, arrhythmias, and sudden cardiac death, that occur despite adherence to Optimal Medical Therapy (OMT) [5]. Indeed, the residual risk of wHF remains significant in the HF population and has been detected in major clinical trials, with percentages ranging from 16.3% to 21.8% [5,6,7].

In this issue, addressing residual risk requires an integrated and personalized approach, and the main strategies include OMT, management of comorbidities, and early recognition of wHF episode.

Particularly in the elderly, the role of frailty, depression, cognitive impairment, nutrition, and goals of care are each uniquely relevant to the implementation and success of medical therapy [8], which often lead to deprescribing, due to the knowledge gap regarding the correct management of these drugs [9].

In fact, comorbidities have a crucial impact on the clinical presentation and prognosis of patients with HF. Among these, cognitive impairment (CoI) is one of the most common. Depending on the diagnostic tools used, the prevalence of CoI in patients with HF ranges between 25% and 75%, both in cross-sectional and longitudinal studies, and is markedly higher compared to the general population [10,11]. Many HF patients present with mild cognitive impairment (MCI), with disturbances in memory, attention, and executive functions [12,13]. The presence of cognitive decline further complicates clinical management, contributing to poorer therapeutic adherence and worsening quality of life and prognosis [14,15]. It is estimated that patients with HF and CoI have a five-fold increased risk of mortality [16].

The link between HF and CoI appears multifactorial. Previous studies suggest that reduced cerebral blood flow, caused by hemodynamic alterations, plays a key role [17,18], while concomitant conditions such as atrial fibrillation, atherosclerosis, and arterial hypertension may also contribute to the onset of cognitive deficits [19].

In addition, mood disorders are frequent in patients with HF. Depression is the most common disorder, with a prevalence that increases with disease severity: it is present in 10% of asymptomatic outpatients and up to 40–70% of hospitalized patients in NYHA class III–IV [20,21]. Anxiety is also very common, and about one in four patients presents with both disorders [22]. These psychiatric comorbidities can exacerbate HF symptoms, contributing to a distorted subjective perception of one’s health status, with a negative impact on well-being and quality of life [23].

Of particular interest, more than 69% of elderly patients with HF have at least two geriatric comorbidities, including functional limitations, and as geriatric conditions increase, mortality increases [24,25]. Furthermore, more than one third of elderly patients with HF are at risk of falls. However, to date we have no data on the potential effect of therapeutic optimization on the risk of falls [26].

In this context, comprehensive geriatric assessment (CGA) represents an integrated and interdisciplinary diagnostic tool useful for analyzing the functional capacities and limitations of elderly patients, taking into account multiple clinical aspects. CGA has been shown to predict mortality, both during hospitalization and in the long term, in elderly patients hospitalized for acute HF [27,28]. In terms of therapeutic innovation, vericiguat represents a relevant novelty. A stimulator of soluble guanylate cyclase (sGC), it acts by enhancing the nitric oxide pathway, which is dysfunctional in patients with HFrEF. In the VICTORIA trial, vericiguat showed a 10% relative risk reduction in the composite endpoint of cardiovascular death or first HF hospitalization (HR 0.90; 95% CI 0.82–0.98; *p* = 0.02), with an NNT of 24 patients, to prevent one event in one year [29]. At the same time, the STRONG-HF study demonstrated that early and intensive optimization of drug therapy (ACEi/ARNI, beta-blockers, MRAs, SGLT2-i) significantly reduced the risk of death and re-hospitalization for heart failure at 180 days (HR 0.66; *p* < 0.001) compared with standard practice [30].

To date, no studies are available that have demonstrated an impact of wHF on cognitive function, risk of falls and comprehensive geriatric assessment in elderly patients with wHF, and the possible role of the vericiguat on CGA scale variations in this population.

Therefore, the aim of this study was to evaluate the effects of treatment with vericiguat on functional domain and risk of falls, cognitive function, humoral, clinical, and echocardiographic variables in a cohort of elderly patients affected by HFrEF with a recent episode of wHF.

## 2. Results

Table 1 reports the comorbidities and medical therapy at baseline of the 55 patients that completed the study. At 12 months, the mean vericiguat dose was 5.5 ± 2.9 mg/day: 18 patients were taking the 2.5 mg/day dose, 22 were taking the intermediate 5 mg/day dose, and 15 were taking the maximum 10 mg/day dose. No severe adverse events were reported during follow-up; there were five mild symptomatic hypotension events, which did not require discontinuation of Vericiguat.

After a 12-month follow-up period, it was possible to observe a clinically and statistically significant improvement in several analyzed variables. The primary endpoint of the study was the change in CGA scales, including MMSE, GDS, SPPB, ADL, IADL, and risk of falls. In addition, possible changes in clinical symptoms, key biochemical parameters, and echocardiographic parameters were also evaluated (Table 2).

First of all, regarding cognitive functions, a statistically significant improvement of the MMSE was observed, increasing from an initial value of 25.1 ± 1.7 to 26.2 ± 2.1 points (*p* < 0.0001). This finding suggests a favorable trend in general cognitive abilities, often considered an indicator of the overall neuropsychological status of the patient.

At the same time, from the emotional point of view, clinically and statistically significant changes were also observed: evaluations carried out using the Geriatric Depression Scale (GDS) documented a reduction in scores, which decreased from a mean value of 7.8 ± 2.0 to 5.4 ± 1.6 points (*p* < 0.0001), showing a relevant benefit in terms of reduction of depressive symptoms and improvement of overall psychological well-being.

From the functional point of view, significant changes were also observed, with variations in the Short Physical Performance Battery (SPPB) from 6.7 ± 1.1 to 8.4 ± 0.9 points (*p* < 0.0001), highlighting an improvement in overall physical performance and basic motor skills, accompanied by an improvement in gait speed from 0.70 ± 0.1 to 0.83 ± 0.06 m/s, a result associated with greater motor autonomy and a reduced risk of disability (*p* < 0.0001).

In association with these changes, we also observed improvements on further geriatric aspects, such as the risk of falls. In fact, from baseline throughout the follow-up, we observed an improvement on the Conley scale, from 5.2 ± 2.3 to 2.4 ± 1.8 pt, *p* < 0.0001.

**Figure 1:**
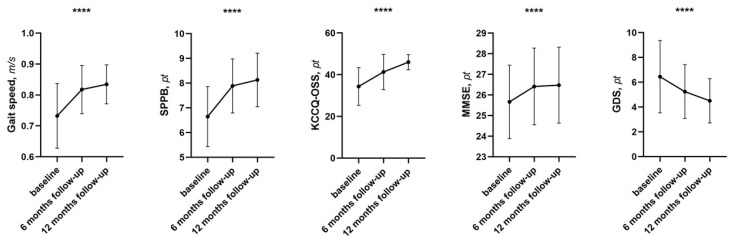
Changes in cognitive and functional parameters between baseline and follow-up. Abbreviations: SPPB: short performance physical battery; KCCQ-OSS: Kansas City cardiomyopathy questionnaire overall summary score; MMSE: Mini-Mental state examination; GDS: Geriatric Depression Scale. **** *p* < 0.0001.

In addition to the results already described, a significant improvement was also detected in clinical symptomatology, as assessed through the Kansas City Cardiomyopathy Questionnaire—Overall Summary Score (KCCQ-OSS). The scores showed a mean reduction from 34.4 ± 8.3 to 46.0 ± 8.3 points (*p* < 0.0001), confirming a benefit perceived by patients in terms of quality of life and reduction of the impact of symptoms related to cardiac disease (Figure 1). Of particular interest, we observe a significant reduction in systemic congestion as demonstrated by the variations in NT-pro-BNP levels from 980 (467–2106) to 654 (274–1762) pg/mL (*p* < 0.0001). Of particular interest, the reduction in systemic congestion, detected clinically and echocardiographically during follow-up, allowed us to reduce the use of loop diuretics from 94.5% of patients to 69.1%, *p* = 0.0005; and anti-aldosteronic drugs from 98.1% of patients to 81.8%, *p* = 0.004.

### 2.1. Echocardiographic Changes

In this contest, echocardiographic parameters also showed statistically significant changes that further reinforce the overall picture of clinical improvement (Table 3). In particular, a favorable increase of Global Longitudinal Strain (GLS) was observed, rising from a mean value of −9.2 ± 1.7 to −11.5 ± 2.1 (*p* = 0.008), suggesting a recovery of left ventricular systolic function. In association, left ventricular filling pressures were also significantly reduced: E/e′ ratio decreased from 16.6 ± 2.4 to 11.9 ± 3.5 (*p* < 0.0001), indicating an improvement of diastolic function and IVC diameter from 20.4 ± 2.3 to 17.0 ± 1.9 (*p* < 0.0001).

In this setting, systolic pulmonary artery pressure (s-PAP) also decreased, from 46.3 ± 6.7 to 41.9 ± 6.2 mmHg (*p* < 0.0001), a finding associated with a lower degree of hemodynamic overload of the pulmonary circulation.

**Figure 2:**
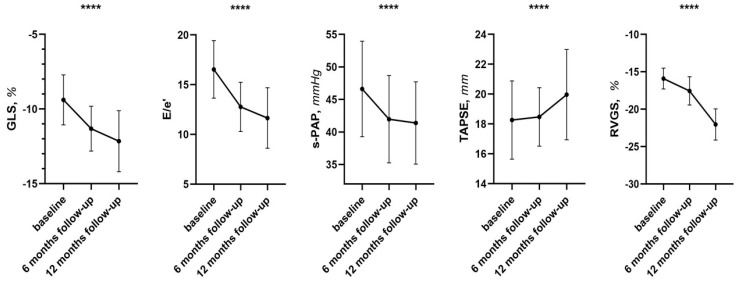
Changes in echocardiographic parameters between baseline and follow-up. Abbreviations: GLS: global longitudinal strain; E/e′: between wave E and wave e′ (reliable estimate of changes in end-diastolic blood pressure); s-PAP: systolic pulmonary arterial pressure; TAPSE: tricuspid annulus plane systolic excursion; RVGS: right ventricular global strain. **** *p* < 0.0001.

In fact, an improvement in right heart function was also observed, with significant variations of tricuspid annular plane systolic excursion (TAPSE), from 17.1 ± 0.07 to 20.0 ± 0.1 mm (*p* < 0.0001), indicating an improvement in right ventricular contractile capacity. This data was confirmed by the improvement of right ventricular global longitudinal strain (RVGS), which showed a favorable increase from −15.9 ± 1.4 to −22.0 ± 2.1 (*p* < 0.0001), and FWS from −18.7 ± 1.6 to 22.9 ± 1.9 (*p* < 0.0001), confirming a more effective systolic function of the right ventricle (Figure 2).

### 2.2. Linear Regression Analysis

The linear correlation analysis was conducted by evaluating the variations of SPPB as the dependent variable, and the variations of different variables under study as independent variables. Predictors were selected a priori based on clinical plausibility. From this model it emerges that the variation of the SPPB score (ΔSPPB) was significantly correlated both with the variation of serum NT-proBNP levels (ΔNT-proBNP) and with the left ventricular ejection fraction (ΔLVEF %). These results suggest that changes in cardiac function and in biochemical markers of heart failure are significantly correlated with variations in functional abilities assessed through SPPB (Table 4).

Subsequently, we constructed a multivariate linear regression model, considering the variation of SPPB as the dependent variable. In this model, the variable that correlated the most with the dependent variable was the variation of left ventricular ejection fraction (ΔLVEF %), which correlated for 27.8% with the variation of SPPB. This finding indicates that an increase in the contractile function of the left ventricle translates into a clinically relevant improvement in physical capacity (Table 5).

Moreover, the variations of circulating NT-proBNP levels (ΔNT-proBNP) also correlated with the variations of SPPB for 6.7%, and the entire model correlated for a total of 34.5%. Therefore, the integration of information related both to ventricular function (ΔLVEF %) and to biochemical markers of myocardial stress (ΔNT-proBNP) allows for a more complete understanding of the determinants of variation in physical performance measured through SPPB in elderly patients affected by chronic heart failure (Table 5).

## 3. Discussion

In this prospective real-world cohort of elderly patients with HFrEF and a recent episode of worsening heart failure, already receiving guideline-directed quadruple therapy, the addition of vericiguat over 12 months was associated with a multidomain improvement in clinical status. NT-proBNP levels decreased and both left and right ventricular structure and function improved, including an increase in LVEF, more favorable left ventricular and right ventricular strain, and lower estimated pulmonary pressures. In parallel, we observed clinically relevant gains in cognition, mood, physical performance, gait speed, and risk of falls, as captured by a comprehensive geriatric assessment. To our knowledge, this is the first study to systematically evaluate the relationship between Vericiguat treatment and the associations with changes in CGA domains in an elderly, highly comorbid HFrEF population after wHF.

The present findings extend and complement the evidence from VICTORIA and VICTOR [29,31]. In VICTORIA, vericiguat reduced the relative risk of the composite of cardiovascular death or first HF hospitalization in a younger, high-risk population with recent wHF, but geriatric outcomes were not assessed. In VICTOR, vericiguat was tested in clinically stable outpatients without recent wHF, again with a focus on hard cardiovascular endpoints. Our cohort differs in several respects: patients were older, more comorbid, and routinely treated with contemporary OMT including SGLT2 inhibitors, and the primary focus was on functional and geriatric trajectories rather than adjudicated HF events [29].

In this context, Vericiguat determined a 10% relative risk reduction of the composite endpoint of cardiovascular death or first hospitalization for heart failure (HR 0.90; 95% CI 0.82–0.98; *p* = 0.02). However, compared to our study, the patients enrolled were of lower mean age (67 years vs. 76.4 years) and only a small percentage were treated with SGLT2i, whereas in our study all patients were taking SGLT2i. Therefore, our study represents further confirmation of the safety of Vericiguat in patients affected by wHF, including elderly patients with multiple comorbidities and on polypharmacy [29].

In particular, by evaluating the results of the VICTOR and VICTORIA studies, it is possible to understand how Vericiguat therapy is influenced by the patient’s risk profile and frailty. While in unstable patients, Vericiguat reduces the risk of wHF, and in compensated patients, it contributes to maintaining clinical stability, in frail elderly patients, it can determine a multidimensional improvement in well-being, including cognitive functions, mood, and quality of life [29,31].

However, in the specific case of our study, the patient pool is older, affected by more comorbidities and has a greater geriatric burden. Furthermore, in the VICTORIA/VICTOR studies, the endpoints were hard (mortality and wHF), while in our study they are mainly functional surrogates related to the patient’s quality of life.

At the real-world level, a direct comparison can be made with a multicenter observational study conducted by Tian et al., in which 200 patients affected by HFrEF, treated with OMT according to guidelines, with or without Vericiguat, were evaluated over a 6-month follow-up. The study showed a significant reduction in NT-proBNP levels in patients treated with Vericiguat, but no structural improvement in left ventricular remodeling [32]. In our study, conducted on elderly patients, therapy with Vericiguat was associated not only with a reduction in circulating NT-proBNP levels, but also with an improvement in echocardiographic parameters, in particular a reduction in left ventricular volume, with a consequent increase in LVEF from 36.8 ± 3.1 to 43.4 ± 5.7% (*p* < 0.001), and improvement in GLS from −9.2 ± 1.7 to −11.5 ± 2.1% (*p* = 0.008). Of particular interest, an improvement in right heart function was also observed, with TAPSE varying from 17.1 ± 0.07 to 20.0 ± 0.1 mm (*p* < 0.001), s-PAP from 46.3 ± 6.7 to 41.9 ± 6.2 mmHg (*p* < 0.001), RVGS from −15.9 ± 1.4 to −22.0 ± 2.1% (*p* < 0.001), and FWS from −18.7 ± 1.6 to −22.9 ± 1.9% (*p* < 0.001).

**Figure 3:**
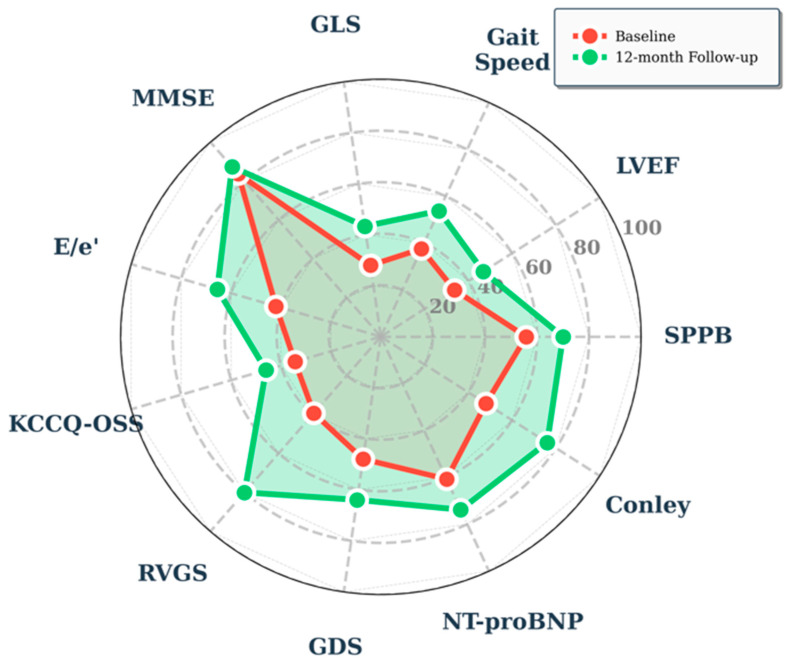
Multidomain improvements with Vericiguat treatment. Spider Plot: All parameters normalized to 0–100 scale. Higher value: better outcomes. Inverted scales for: GLS, RVGS (more negative = better); Conley, GDS, NTproBNP, E/e′ (lower = better). Abbreviations: NT-proBNP: N-terminal pro-brain natriuretic peptide; MMSE: Mini mental state examination; GDS: Geriatric Depression Scale; SPPB: Short Physical performance battery; KCCQ-OSS: Kansas City Cardiomyopathy Questionnaire—Overall Summary Score; GLS: Global Longitudinal strain; RVGS: right ventricular global longitudinal strain; LVEF: Left ventricular ejection fraction.

However, the most fundamental aspect of our study was the evaluation of the variations in CGA parameters between baseline and follow-up, in addition to the classical clinical and echocardiographic parameters. Indeed, we observed improvements in cognitive functions (MMSE from 25.1 ± 1.7 to 26.2 ± 2.1 points, *p* < 0.0001), mood (GDS from 7.8 ± 2.0 to 5.4 ± 1.6 points, *p* < 0.0001), physical performance (SPPB from 6.7 ± 1.1 to 8.4 ± 0.9 points, *p* < 0.0001), gait speed (from 0.70 ± 0.1 to 0.83 ± 0.06 m/s, *p* < 0.0001), and quality of life (KCCQ from 34.4 ± 8.3 to 46.0 ± 8.3 points, *p* < 0.0001) (see Spider plot, Figure 3). Of particular interest, in our study, we observed an improvement in Conley’s scale score (from 5.2 ± 2.3 to 2.4 ± 1.8 pt, *p* < 0.0001) assessed at baseline and during follow-up, which translates into a reduced risk of falls, a key aspect in the management of elderly patients with HF. A possible pathophysiological explanation for these variations could be due to the improvement in cardiac output and, consequently, in clinical symptoms and quality of life, probably through better cerebral and systemic perfusion. In fact, in the absence of a control group, it is not possible to attribute the improvements (especially cognitive/biochemical) with certainty to treatment with Vericiguat, but this is a descriptive assessment.

In this context, several studies have shown that optimization of HF therapy is associated with improvement in CGA scales, but these are often studies conducted on patients with chronic HF without a worsening episode, not on quadruple therapy (as in our study), and for a total follow-up of 6 months (and not 12, as in our study) [32,33]. These results are even more relevant considering that in the elderly population not all patients receive the pharmacological classes recommended by guidelines, and that only a small percentage of patients reach the target dose of 10 mg/day for Vericiguat. In fact, a German retrospective study conducted on 2916 patients with a mean age of 73 years treated with Vericiguat demonstrated high therapy adherence, but only about 36% of patients reached the target dose of 10 mg. In particular, women and elderly patients less frequently achieved the 10 mg dose. The initiation of Vericiguat treatment was also associated with an increase in the adoption of quadruple therapy (from 29% to 44%), suggesting that the drug may act as a catalyst in optimizing therapy [34].

Finally, a recent prospective Chinese study by Zhao et al. evaluated the efficacy of Vericiguat in association with the four pillars of HFrEF treatment (ARNI, beta-blockers, MRA, SGLT2). A total of 103 patients were enrolled, with a mean age of 59.02 ± 12.55 years, affected by HF divided into subgroups based on etiology (post-MI, CMD, VHD) and ejection fraction (HFpEF 10.68%, HFmrEF 28.16%, HFrEF 61.17%), most of them in NYHA class II–III. After one month of treatment, patients with HFrEF showed a significant improvement in LVEF% (from 38% to 43%), a reduction in NT-proBNP (from 4567 to 1895 ng/L), and a significant improvement in quality of life, as assessed by a reduction in MLHFQ score from 45.7 to 32.3 points (*p* < 0.01), without alterations in liver or renal function. Despite these encouraging results, this was a study conducted mainly on younger patients with a short follow-up, unlike our study with a 12-month follow-up [35].

In line with the literature, our work highlights that an integrated therapeutic approach, including both innovative drugs such as Vericiguat and multidimensional assessment tools, can allow better management of elderly patients affected by this condition. It is known, in fact, that despite the adoption of cornerstone therapies (ACEi/ARNI, beta-blockers, MRA, SGLT2 inhibitors), a residual risk of rehospitalization and mortality persists. In the PARADIGM-HF trial, for example, 21.8% of patients treated with sacubitril/valsartan had a new hospitalization for HF during a 27-month follow-up; in DAPA-HF, despite dapagliflozin, the event rate remained 16.3%; and in EMPEROR-Reduced, empagliflozin reduced but did not eliminate the risk (19% hospitalizations).

In this context, Vericiguat emerges as an additional option to address residual risk, especially in frail elderly patients with wHF episodes. The combination of Vericiguat with early optimization of therapy, as demonstrated by the STRONG-HF study (HR 0.66; *p* < 0.001) and as confirmed by the real-world experiences of Kerwagen et al. and Zhao et al., could represent a winning strategy to further reduce mortality and rehospitalizations in a particularly vulnerable phenotype such as the elderly [36].

A peculiar aspect of our study concerns the integration of pharmacological therapy and multidimensional geriatric assessment. The CGA has already been shown to predict mortality and clinical outcomes in elderly patients hospitalized for acute HF [16]. However, no data were previously available on the impact of Vericiguat treatment on cognitive and functional domains. Our results suggest that the optimization of hemodynamic and left ventricular function may translate into improvements in cognitive abilities (MMSE), mood (GDS), and quality of life (KCCQ). Of particular interest, in our study, we observed a significant improvement in functional abilities measured by the SPPB, which was associated with a significant improvement in gait speed and in the risk of falls assessed by the Conley scale. This aspect is fundamental if we consider that in elderly patients suffering from HF, in addition to dyspnea and the presence of oedemas, there may be associated alterations in posture control, which when associated with other comorbidities such as anaemia, visual or hearing impairment, and polypharmacotherapy, increase the risk of falls, with a negative effect on prognosis [37].

In fact, a reduction in the risk of falling (documented using the Conley scale) combined with an improvement in walking speed can reduce not only “geriatric-centered” outcomes but also potentially hospitalizations and disabilities.

This study presents some limitations: first of all, it is a monocentric investigation, not configured as a randomized clinical trial, and lacking a matched control group. However, each patient can be considered as their own control, since before enrollment they had already been treated with the best available therapy according to current guidelines, while remaining symptomatic with an episode of wHF. Further limitations are represented by the relatively small sample size, limited to elderly patients with HFrEF and recent wHF followed in geriatrics, and the short follow-up period. Finally, the following should be taken into consideration: absence of randomization and a control group, absence of data on nutritional aspects and physiotherapy, wHF, possible selection bias (motivated patients, followed in a specialist setting), and potential confounding factors (intensive geriatric and cardiological follow-up for 12 months; concomitant optimization of other medications, such as reduction of diuretics, adjustment of ACEi/ARNI).

## 4. Materials and Methods

### 4.1. Study Population

All patients included in the study had a baseline diagnosis of wHF, understood as HFrEF/HFmrEF (NYHA II-III) in OMT, and manifested a worsening of symptoms requiring intensification of diuretic therapy with recourse to e.v. diuretic therapy, either as an outpatient or as an ordinary inpatient. Vericiguat was introduced into therapy after hemodynamic stabilization, after an average period of 8 ± 2.5 days. Patients eligible for Vericiguat, in addition to their previous therapy, received initial dosage of 2.5 mg/die; the dosage was increased every 2–4 weeks up to the maximum tolerated dose.

Exclusion criteria were: chronic kidney disease stage IV K-DOQI (eGFR < 30 mL/min/1.73 m^2^, CKD-EPI), severe hepatic impairment (Child-Pugh Class C), history of angioedema, previous diagnosis of dementia or serious psychiatric disorders.

At the enrollment visit and at subsequent follow-ups at 6 and 12 months, medical history data were collected, and the following were performed: complete physical examination for the assessment of the main clinical parameters, determination of the NYHA functional class, and evaluation of quality of life through the Minnesota Living with Heart Failure Questionnaire (MLHFQ). Anthropometric parameters (weight, height, BMI) were measured. Twelve-lead ECGs, blood tests, and color-Doppler echocardiography with speckle tracking method were performed.

From an initial cohort of 67 patients, five were excluded for the presence of psychiatric disorders, four for the presence of CKD, and one for the presence of dementia, so 55 patients (45 men and 10 women), with a mean age of 76.4 ± 5.1 years, attending the outpatient clinic dedicated to heart failure of the Geriatrics Unit at the University Hospital “Renato Dulbecco” of Catanzaro, affected by HFrEF (NYHA II-III), and already on optimized medical therapy who developed an episode of wHF, were enrolled.

### 4.2. Comprehensive Geriatric Assessment (CGA)

CGA was performed at the time of inclusion, repeated at 6 and 12 months of follow-up, and included the following tools: Mini-Mental State Examination (MMSE): the most commonly used neuropsychological tests for assessing cognitive functions [38]; Activities of Daily Living (ADL): scale that assesses patient’s ability to handle basic self-care tasks [39,40]; Geriatric Depression Scale—Short Form (GDS-S): a 15-item self-report assessment used to identify depression in elderly [41]. Short Physical Performance Battery (SPPB): test for the evaluation of lower limb function [42]. In addition, the risk of falling was assessed by performing the Conley scale [43] (see Supplementary File). CGA was performed by a single trained operator, who was blinded to treatment protocol.

In addition, the walking test was performed to evaluate gait speed, an important clinical parameter to assess general health status, physical function, and patient prognosis. It is measured by having the patient walk along a straight 4 m path and timing the duration, with the result expressed in meters/second (m/s). A speed < 0.6 m/s is associated with high risk of mortality and disability, while values > 1.2 m/s are considered normal.

### 4.3. Blood Pressure Measurement

Blood pressure (BP) was measured at the non-dominant arm, with the patient in the supine position after 5 min of rest. At least three measurements were taken on different occasions, about two weeks apart. Systolic blood pressure (SBP) and diastolic blood pressure (DBP) were recorded at the first (phase I) and last (phase V) Korotkoff sounds, respectively. Baseline BP values correspond to the average of three measurements taken at three-minute intervals [44].

### 4.4. Color-Doppler Echocardiography

Echocardiographic examinations were performed with the VIVID E-95 system (GE Technologies, Milwaukee, WI, USA) and a 2.5 MHz probe, with the patient in the left lateral decubitus position and at rest, according to the recommendations of the American Society of Echocardiography, minimizing the depth in order to optimize the frame rate (40–80 fps) [45]. All evaluations were conducted by the same experienced operator, using preferably the apical approach for volumetric measurement of cardiac chambers.

Using Simpson’s method, the following were calculated: left ventricular end-diastolic volume (LVEDV) and end-systolic volume (LVESV), expressed in mL and indexed to body surface area (LVEDV/BSA and LVESV/BSA, mL/m^2^). The ejection fraction (LVEF) was calculated as: LVEF = (LVEDV − LVESV)/LVEDV × 100. Left atrial volume (LAVI) was measured using the area-length method and indexed to BSA (LAVi, mL/m^2^).

Right ventricular systolic function was evaluated by tricuspid annular plane systolic excursion (TAPSE) and estimation of systolic pulmonary artery pressure (s-PAP). The latter was calculated from the peak tricuspid regurgitation velocity (TRV peak) using the Bernoulli equation: s-PAP = 4 (TRV peak)^2^ + right atrial pressure. The latter was estimated from the diameter and collapsibility of the inferior vena cava (IVC). Right ventricular outflow tract (RVOT) diameter and right atrial area were also measured.

Diastolic function was assessed with pulsed Doppler at the level of the mitral leaflets to obtain E and A waves (m/s) and the E/A ratio, and with Tissue Doppler at the level of the lateral mitral annulus for velocities e′ and a′ (m/s) and the E/e′ ratio, indicative of left ventricular filling pressures [46]. Using dedicated software, global longitudinal strain (GLS) was analyzed. Right ventricular global strain (RVGS) was assessed using the RV-focused apical four-chamber view, analyzing the free wall segments, and free wall strain (FWS) of the right ventricle analyzing the free wall of the right ventricle. GWE was calculated using dedicated software that incorporates strain data and blood pressure measurements [47]. For speckle tracking analysis digital loops were captured, recording at least three consecutive beats, and analyzed off-line using a dedicated software (EchoPAC 20.0; GE Medical Systems, Milwaukee, United States) by two operators who were blinded to the clinical characteristics of the patients.

### 4.5. Statistical Analysis

Data were expressed as mean and standard deviation (mean ± SD), for normally distributed data, as median and interquartile range (IQR) for data not normally distributed, and as number and percentage (%) for categorical variables. Changes between baseline and the two follow-ups (6 and 12 months) were analyzed using ANOVA test for paired data, and chi-square tests for categorical variables. A *p*-value < 0.05 was considered statistically significant. A simple linear regression analysis was performed to assess the correlation between variation of SPPB, expressed as ∆ of variation between baseline and follow-up (∆T0–12), and the variation of several comorbidities also expressed as ∆T0–12. Variables that reached statistical significance were entered into a stepwise multivariate linear regression model to evaluate the magnitude of their individual effect on ∆SPPB. All analyses were performed using the SPSS 20.0 statistical program for Windows (SPSS Inc., Chicago, IL, USA).

## 5. Conclusions

This study provides evidence that the addition of Vericiguat to the medical therapy in patients with HFrEF, with recent episode of worsening, was associated with a positive effect on cognitive, functional, and psychological domains. In particular, in this study, we observed a significant improvement in functional abilities, gait speed, and risk of falls.

Further prospective and randomized studies focusing on geriatric populations will be necessary to confirm these results and integrate CGA into clinical pathways for heart failure.

## Figures and Tables

**Table 1:** Comorbidities and drugs therapy of the study population at the baseline.

Male/Female sex, *n* (%)	45/10 (81/19)
Age, years	76.4 ± 5.1
IHD, *n* (%)	34 (62)
VHD, *n* (%)	23 (41)
AF, *n* (%)	24 (43)
T2DM, *n* (%)	33 (60)
AH, *n* (%)	48 (87)
COPD, *n* (%)	21 (38)
Dyslipidaemia, *n* (%)	44 (80)
CKD, *n* (%)	25 (45)
MRAs, *n* (%)	45 (81)
Statins, *n* (%)	49 (89)
β-blockers, *n* (%)	51 (92)
DOACs, *n* (%)	24 (43)
Anti-platelet drugs, *n* (%)	28 (50)
Diuretics, *n* (%)	52 (94)
OADs, *n* (%)	29 (52)
SGLT2i, *n* (%)	47 (85)
ACEi/ARNI, *n* (%)	45 (81)

Abbreviations: IHD: Ischemic Heart Disease; VHD: Valvular Heart Disease; AF: atrial fibrillation; T2DM: Diabetes Mellitus Type 2; AH: Arterial Hypertension; COPD: chronic obstructive pulmonary disease; CKD: chronic kidney disease; MRAs: mineralocorticoid receptor antagonist; DOACs: Oral anti-coagulants; OADs: oral antidiabetic drugs; SGLT2i: sodium glucose cotransporter 2 inhibitors; ACEi: Angiotensin-converting enzyme inhibitors; ARNI: Angiotensin Receptor-Neprilysin Inhibitor.

**Table 2:** Clinical characteristics of the study population.

	Baseline	6-Months Follow-Up	12-Months Follow-Up	*p* Value *
Gait Speed, m/s	0.70 ± 0.1	0.81 ± 0.07	0.83 ± 0.06	<0.0001
MMSE, pt	25.1 ± 1.7	26.3 ± 2.2	26.2 ± 2.1	<0.0001
GDS, pt	7.8 ± 2.0	6.1 ± 1.6	5.4 ± 1.6	<0.0001
IADL, pt	5.9 ± 1	5.9 ± 1	6.0 ± 1	<0.0001
ADL, pt	4.1 ± 0.7	4.5 ± 0.8	4.7 ± 0.8	<0.0001
SPPB, pt	6.7 ± 1.1	7.4 ± 0.8	8.4 ± 0.9	<0.0001
Conley scale, pt	5.2 ± 2.3	4.0 ± 2.0	2.4 ± 1.8	<0.0001
MLHFQ, pt	85 ± 5	81.7 ± 4.9	80.7 ± 4.7	<0.0001
KCCQ-OSS, pt	34.4 ± 8.3	41.3 ± 10	46.0 ± 8.3	<0.0001
BMI, Kg/m^2^	30.20 ± 5	29.4 ± 4.4	26.60 ± 4.4	<0.0001
SBP, mmHg	119.8 ± 12.6	114.8 ± 10.3	121.1 ± 13.8	<0.0001
DBP, mmHg	68.7 ± 6.1	67.4 ± 6.1	67 ± 9.4	<0.0001
PP, mmHg	51 ± 13	47.3 ± 10	53.7 ± 10	<0.0001
HR, bpm	70.9 ± 8.7	69 ± 8.7	67.5 ± 8.5	0.137
RR, afm	16 ± 2.2	14.8 ± 2.2	16.9 ± 1.4	<0.0001
HB, g/dL	13.3 ± 2	11 ± 1	12.8 ± 2.7	<0.0001
HCT, %	40.1 ± 4.5	43.4 ± 3.5	42.5 ± 7	0.004
RBC, mL 10^3^/uL	4.4 ± 0.5	4.5 ± 0.5	4.7 ± 0.8	<0.0001
WBC, mL 10^3^/uL	6.1 ± 1.1	6.3 ± 1.2	6.9 ± 1.3	<0.0001
PLT, 10^3^/uL	192.1 ± 47.5	186.6 ± 45.6	200.2 ± 57.1	<0.0001
Na, mmol/L	139.9 ± 2	138.8 ± 2.1	143.4 ± 17	<0.0001
K, mmol/L	4.6 ± 0.4	4.7 ± 0.4	4.7 ± 0.6	<0.0001
Glycemia, mg/dL	108.2 ± 26.5	96.8 ± 7.7	106 ± 16.7	0.003
LDL, mg/dL	74.9 ± 31.5	69 ± 23.4	61.3 ± 21.2	0.137
HDL, mg/dL	43.9 ± 11.1	44.9 ± 13	45.9 ± 8.7	0.025
Triglycerides, mg/dL	124.5 ± 53.5	107.8 ± 30.6	110.1 ± 29.7	<0.0001
Creatinine, mg/dL	1.1 ± 0.3	0.96 ± 0.2	1.1 ± 0.5	<0.0001
eGFR, mL/min/1.73 m^2^	66.9 ± 18.3	77.1 ± 19.3	69.6 ± 24.1	<0.018
NTproBNP, pg/mL	980 (467–2106)	785 (320–1550)	654 (274–1762)	<0.0001
Uric acid, mg/dL	5.9 ± 1.2	5.8 ± 1.2	4.8 ± 1.3	<0.0001
Iron, mcg/dL	65 ± 25.1	76 ± 22.2	85.2 ± 21.6	<0.0001
Transferrin, mg/dL	2.2 ± 0.2	2.1 ± 0.3	2.2 ± 0.2	0.562
Ferritin, ng/mL	178.1 ± 117.9	206.9 ± 101	224.3 ± 101.2	<0.0001
hs-CRP, pg/mL	6.6 ± 0.4	6 ± 0.4	4.7 ± 1.4	<0.0001

* Performed by ANOVA for repeated measurements. Abbreviations: MMSE: Mini Mental State Examination; GDS: Geriatric Depression Scale; IADL: Instrumental Activities of Daily Living; ADL: Activities of Daily Living; SPPB: Short Physical Performance Battery; MLHQS: Minnesota Living with Heart Failure Questionnaire score; BMI: Body Mass Index; SBD: Systolic Blood Pressure; DBP: Diastolic Blood Pressure; PP: Pulse Pressure; HR:Heart Rate; RR: Respiratory Rate; HB: Hemoglobin; HCT: Hematocrit; RBC: Red Blood Cells; WBC: White Blood Cells; PLT: Platelets; NA: Sodium; K: Potassium; LDL: Low Density Lipoprotein; HDL: High Density Lipoprotein; eGFR: Estimated Glomerular Filtration Rate; NTproBNP: Pro B-type Natriuretic Peptide; hs-CRP: High Sensitivity-C-Reactive Protein.

**Table 3:** Echocardiographic characteristics of the study population.

	Baseline	6-Month Follow-Up	12-Month Follow-Up	*p* Value *
LAVi, mL/m^2^	48.2 ± 12.1	39.3 ± 9.2	36.7 ± 10.3	0.409
LVEDV/BSA, mL/m^2^	87.9 ± 8.9	83.8 ± 9.9	83.1 ± 21.6	<0.0001
LVESV/BSA, mL/m^2^	57.4 ± 6.2	53.3 ± 5.7	53 ± 5.5	0.003
E/A	0.64 ± 0.1	0.72 ± 0.1	0.70 ± 0.09	0.003
E/e′	16.6 ± 2.4	12.4 ± 1.7	11.9 ± 3.5	<0.0001
LVEF, %	36.8 ± 3.1	37.2 ± 5.1	43.4 ± 5.7	<0.0001
GLS, %	−9.2 ± 1.7	−11.3 ± 1.5	−11.5 ± 2.1	0.008
GWE, %	86.7 ± 3.2	92.1 ± 2.5	90.3 ± 5.2	0.045
RVGS, %	−15.9 ± 1.4	−17.6 ± 1.9	−22.0 ± 2.1	<0.0001
FWS, %	−18.7 ± 1.6	−20.4 ± 1.7	−22.9 ± 1.9	<0.0001
RVOTp, cm	2.5 ± 0.3	2.1 ± 0.2	2.2 ± 0.5	<0.0001
RAA, cm^2^	19.5 ± 2.1	17.5 ± 2.6	16.6 ± 1.9	0.010
TAPSE, mm	17.1 ± 0.07	17.9 ± 0.08	20.0 ± 0.1	<0.0001
s-PAP, mmHg	46.3 ± 6.7	42 ± 6.9	41.9 ± 6.2	<0.0001
TAPSE/s-PAP, mm/mmHg	0.39 ± 0.07	0.44 ± 0.08	0.47 ± 0.1	<0.0001
IVC, mm	20.4 ± 2.3	18.7 ± 1.4	17 ± 1.9	<0.0001

* Performed by ANOVA for repeated measurements. Abbreviations: LAVi: Left Atrial Volume Index; LVEDV/BSA: left ventricular end-systolic volume/body surface area; LVESV/BSA: left ventricular end-systolic volume/body surface area; E/A: marker of diastolic dysfunction; E/e′: ventricular filling marker; LVEF: Left Ventricular Ejection Fraction; GLS, global longitudinal strain; GWE: Global Work Efficiency; RVGS: right ventricular global strain; FWS: Free Wall Strain; RVOTp, right ventricular outflow tract; RAA: Right Atrium area; TAPSE: Tricuspid Annulus Plane Systolic Excursion; s-PAPS: Systolic Pulmonary Arterial Pressure; IVC: inferior vena cava.

**Table 4:** Simple linear correlation analysis between Δ of SPPB and Δ of different covariates in the study population.

	ΔSPPB R/P
ΔMMSE	0.134/0.304
ΔGDS	−0.097/0.464
ΔNT pro BNP, pg/dL	−0.265/0.049
Δhs-CRP, mg/L	−0.50/0.747
ΔLVEF, %	0.331/0.043
ΔGLS, %	−0.175/0.162
ΔE/e′	−0.139/0.260

Abbreviations: Δ: variation between baseline and twenty-months follow-up, MMSE: Mini Mental state examination; GDS: Geriatric Depression Scale; SPPB: short performance physical battery; NT-pro-BNP: Pro B-type Natriuretic Peptide; hs-CRP: high sensitivity C-reactive protein; LVEF: Left Ventricular Ejection Fraction; GLS: global longitudinal strain; E/e′: between wave E and wave e′ (reliable estimate of changes in end-diastolic blood pressure).

**Table 5:** Stepwise multivariate linear regression analysis between Δ of SPPB as the dependent variable and Δ of different covariates.

Δ of SPPB as Dependent Variable			
All	R2 Partial	R2 Total	*p*
ΔLVEF, %	27.8%	27.8%	<0.001
ΔNT pro BNP, pg/dL	6.7%	34.5%	<0.001

Abbreviations: Δ: variation between baseline and 20-month follow-up; SPPB: short performance physical battery; NT-pro-BNP: Pro B-type Natriuretic Peptide; LVEF: Left Ventricular Ejection Fraction.

## Data Availability

The original contributions presented in this study are included in the article/Supplementary Materials. Further inquiries can be directed to the corresponding author.

## Supplementary Materials

The following supporting information can be downloaded at: https://www.mdpi.com/article/10.3390/ph19030466/s1.

## Abbreviations

The following abbreviations are used in this manuscript:

HFrEF	Heart failure with reduced ejection fraction
wHF	Worsening Heart Failure
OMT	optimal medical therapy
CGA	Comprehensive geriatric assessment
HF	Heart failure
NT-proBNP	N-terminal pro-brain natriuretic peptide
MMSE	Mini mental state examination
GDS	Geriatric Depression Scale
SPPB	Short Physical performance battery
ACE	Angiotensin Converting Enzyme
MRAs	mineralocorticoid receptor antagonists
ARNI	Angiotensin Receptor–Neprilysin Inhibitor
SGLT2i	sodium-glucose cotransporter-2 inhibitors
CoI	Cognitive Impairment
NYHA	New York Heart Association
sGC	soluble guanylate cyclase
HR	Hazard Ratio
NNT	Number needed to treat
ADL	Activities of Daily Living
IADL	Instrumental Activities of Daily Living
KCCQ-OSS	Kansas City Cardiomyopathy Questionnaire—Overall Summary Score
GLS	Global Longitudinal strain
IVC	Inferior vena cava
s-PAP	systolic pulmonary artery pressure
TAPSE	tricuspid annular plane systolic excursion
RVGS	right ventricular global longitudinal strain
FWS	Free wall strain
LVEF	Left ventricular ejection fraction
