# Supplementary material for: Association of Vericiguat with Improvement in Functional Abilities and Comprehensive Geriatric Assessment in Elderly Patients with Worsening Heart Failure
Source: Pharmaceuticals (Basel). 2026 Mar 12;19(3):466. doi: 10.3390/ph19030466 (PMC13029400; doi:10.3390/ph19030466)
Supplement: Supplementary file 1 [file pharmaceuticals-19-00466-s001.zip › pharmaceuticals-4175825-supplementary.pdf]

SUPPLEMENTARY

Mini-Mental State Examination (MMSE): test for the evaluation of cognitive disorders, consisting of 30 items investigating seven areas: temporal orientation, spatial orientation, word registration, attention and calculation, recall, language, and constructive praxis. The total score ranges from 0 to 30. A score ≤18 indicates severe cognitive impairment; a score between 18 and 24 indicates mild/moderate cognitive impairment; a score of 25 is considered borderline; a score between 26 and 30 is indicative of normal cognition [29].

Activities of Daily Living (ADL): scale consisting of six basic activities (bathing, dressing, toileting, transferring, continence, feeding), scored from 0/6 (maximum dependence) to 6/6 (maximum independence) [30].

Instrumental Activities of Daily Living (IADL), or Lawton scale: evaluates autonomy in eight instrumental activities. Each activity is scored 1 point if performed independently or 0 in case of dependence. The total score ranges from 0 (total dependence) to 8 (complete independence) [31].

Geriatric Depression Scale – Short Form (GDS-S): short version consisting of 15 items. Ten items indicate depression if answered affirmatively, while five do so if answered negatively. A score >5 suggests the need for further investigation; a score ≥10 is strongly indicative of depression [32].

Short Physical Performance Battery (SPPB): test for the evaluation of lower limb function, structured into three sections: Static balance in three positions (feet together, semi-tandem, tandem) maintained for 10 seconds each. The score ranges from 0 (unable to maintain the basic position) to 4 (complete execution); 4-meter walk: the time taken determines the score of the section; Repeated chair stands: the patient must stand up five times consecutively without using the arms. The score ranges from 0 (unable to complete the test) to 4 (completion in less than 11.2 seconds). The total score ranges from 0 (maximum disability) to 12 (maximum performance) [33].

In addition, the risk of falling was assessed by performing the Conley scale. This scale consists of 2 main parts that assess previous falls and the presence of cognitive decline, for a total of 6 items; a score greater than or equal to 2 indicates an increased risk of falling [34].
